# Supplementary material for: Using virtual reality to study preference and restorativeness for streetscape vegetation design
Source: PLoS One. 2025 Sep 17;20(9):e0332493. doi: 10.1371/journal.pone.0332493 (PMC12443275; doi:10.1371/journal.pone.0332493)
Supplement: Attachment — Submitted filename: Response_Letter.pdf [file pone.0332493.s004.pdf]

Response letter for paper resubmission

Dear Dr. Shu,

On behalf of my co-authors, I am writing to submit a second revised version of our paper, entitled "Using virtual reality to study preference and restorativeness for streetscape vegetation design" (manuscript PONE-D-25-01612R1), for consideration by the PLOS ONE journal.

We would like to reiterate our profound gratitude to you and the reviewers for your invaluable time and effort in reviewing our manuscript.

The adjustments have been made in light of the remaining minor issues that were brought to our attention by the reviewer.

The revised manuscript has been reviewed and approved by all authors, and it is hereby submitted to the editorial board of PLOS ONE for consideration. We eagerly anticipate the eventual formal acceptance of our manuscript.

Sincerely,

Pooria Baniadam, PhD
AAU CRENAU, CNRS, Ecole Centrale de Nantes

Comment	Response
**Reviewer #3**	
1. While the authors provided basic demographic information, it remains unclear what academic or disciplinary backgrounds the participants had. This is particularly relevant given the use of theoretical constructs such as the ART dimensions. Were participants from relevant fields? Were any measures used to ensure participants fully understood the meaning of the four ART-based statements?	We appreciate the reviewer's comment. Participants in this study were recruited from varied backgrounds. We did not restrict recruitment to specific disciplines, as our goal was to capture a general population sample rather than a group with specialized expertise in environmental psychology or urban studies.<br><br>Regarding comprehension of the ART-based statements, no additional training or explanatory measures beyond the statements themselves were provided. This choice was deliberate: we sought to avoid biasing participants' responses by overexplaining or leading them toward particular interpretations. Instead, we used concise statements derived from the validated Perceived Restorativeness Scale (PRS), which are designed to be understandable by laypersons. We acknowledge that interpretations may vary, and we now highlight this as a limitation of the study in the Discussion (Section 4.6, lines 745-750). Future studies could include a brief comprehension check or provide non-technical examples of each dimension to ensure more consistent understanding across participants.
2. The standalone section on "Key Terms" feels disjointed from the overall narrative. I suggest integrating these definitions directly into the literature review, where the terms can be introduced in context.	In the revised manuscript, we have removed the standalone "Key Terms" section. Instead, the definitions of the relevant terms have been integrated into the Background (section 1.1) where these concepts are first discussed. This restructuring improves the flow of the Introduction and ensures that key terms are introduced in direct relation to the studies and theoretical frameworks in which they are applied.
